# Untargeted metabolic analysis in dried blood spots reveals metabolic signature in 22q11.2 deletion syndrome

**DOI:** 10.1038/s41398-022-01859-4

**Published:** 2022-03-09

**Authors:** Dorinde Korteling, Marco P. Boks, Ania M. Fiksinski, Ilja N. van Hoek, Jacob A. S. Vorstman, Nanda M. Verhoeven-Duif, Judith J. M. Jans, Janneke R. Zinkstok

**Affiliations:** 1 Department of Psychiatry and Brain Center, University Medical Center Utrecht, Utrecht, The Netherlands; 2 Department of Pediatrics, Wilhelmina Children’s Hospital, University Medical Center Utrecht, Utrecht, The Netherlands; 3 Department of Psychiatry and Neuropsychology, Division of Mental Health, MHeNS, Maastricht University, Maastricht, The Netherlands; 4 Section Metabolic Diagnostics, Department of Genetics, University Medical Center Utrecht, Utrecht, The Netherlands; 5 Program in Genetics and Genome Biology, Research Institute, The Hospital for Sick Children, Toronto, ON Canada; 6 Department of Psychiatry, University of Toronto, Toronto, ON Canada; 7 Department of Psychiatry, Radboud University Medical Center, Nijmegen, The Netherlands; 8 Karakter Child and Adolescent Psychiatry, Nijmegen, The Netherlands

**Keywords:** Autism spectrum disorders, Clinical genetics, Human behaviour, Diagnostic markers

## Abstract

The 22q11.2 deletion syndrome (22q11.2DS) is characterized by a well-defined microdeletion and is associated with increased risk of neurodevelopmental phenotypes including autism spectrum disorders (ASD) and intellectual impairment. The typically deleted region in 22q11.2DS contains multiple genes with the potential of altering metabolism. Deficits in metabolic processes during early brain development may help explain the increased prevalence of neurodevelopmental phenotypes seen in 22q11.2DS. However, relatively little is known about the metabolic impact of the 22q11.2 deletion, while such insight may lead to increased understanding of the etiology. We performed untargeted metabolic analysis in a large sample of dried blood spots derived from 49 22q11.2DS patients and 87 controls, to identify a metabolic signature for 22q11.2DS. We also examined trait-specific metabolomic patterns within 22q11.2DS patients, focusing on intelligence (intelligence quotient, IQ) and ASD. We used the Boruta algorithm to select metabolites distinguishing patients from controls, patients with ASD from patients without, and patients with an IQ score in the lowest range from patients with an IQ score in the highest range. The relevance of the selected metabolites was visualized with principal component score plots, after which random forest analysis and logistic regression were used to measure predictive performance of the selected metabolites. Analysis yielded a distinct metabolic signature for 22q11.2DS as compared to controls, and trait-specific (IQ and ASD) metabolomic patterns within 22q11.2DS patients. The metabolic characteristics of 22q11.2DS provide insights in biological mechanisms underlying the neurodevelopmental phenotype and may ultimately aid in identifying novel therapeutic targets for patients with developmental disorders.

## Introduction

Over the last few decades an increasing proportion of developmental disorders have been connected to a known genetic etiology [1, 2]. However, the mechanisms relating genetic mutation to phenotypic manifestation are oftentimes poorly understood. Knowledge about these mechanisms would be valuable as this may provide novel therapeutic targets and might aid in symptom prediction and disease stratification. Various genetic disorders may function as a window into the underlying neurobiological pathways that result in the manifestation of developmental disorders. The 22q11.2 deletion syndrome (22q11.2DS) is one such pathogenic genetic variant.

22q11.2DS results from a hemizygous deletion of the long arm of chromosome 22 [3]. The 22q11.2 deletion has an estimated prevalence of 1 in ~2000 live births [4] and is associated with a highly variable clinical presentation, affecting multiple organs and tissues. The clinical phenotype may include heart anomalies, palatal abnormalities, facial dysmorphisms, T-cell abnormalities and endocrine gastrointestinal problems [5, 6]. Furthermore, 22q11.2DS is associated with cognitive deficits and neurodevelopmental symptoms [3]. Most of the genes that are typically deleted in 22q11.2DS are expressed in the brain [7]. Patients with 22q11.2DS have an increased risk of developing brain-related phenotypes, including language impairment, anxiety disorders, attention-deficit hyperactivity disorder and autism spectrum disorder (ASD) in early life, as well as schizophrenia and early onset Parkinson’s disease later in life [6, 8–12].

Intellectual disability is common in people with 22q11.2DS, with an estimated prevalence of 45–50% [9]. The mean Intelligence Quotient (IQ) in individuals with 22q11.2DS is ~70, with approximately two-third of the population having an IQ in the range of 55–85 [13], as opposed to a mean IQ of 100 in the general population. Furthermore, ASD is highly prevalent among individuals with 22q11.2DS. The prevalence of ASD in 22q11.2DS patients has been estimated to be around 35% [9, 14].

Over the last few decades, 22q11.2DS has been well characterized genetically [3]. However, despite the fact that 90% of people with 22q11.2DS carry an identical mutation [3], the clinical phenotype is highly heterogeneous. The reasons for this phenotypic variability remain largely unclear and may include epigenetic mechanisms, genetic risk variants outside the 22q11.2 locus and environmental factors. Recently, progress has been made towards elucidating the mechanisms behind this phenotypic variability, illustrating the role of common genetic variation and parental phenotypes [15, 16].

One relevant but understudied mechanism behind phenotypic variability in 22q11.2DS is metabolic functioning [3, 17]. Out of the ~90 genes involved in 22q11.2 DS, nine genes are implicated in key metabolic processes: Catechol-O-Methyltransferase (*COMT*), Ubiquitin Recognition Factor In ER-Associated Degradation 1 (*UFD1L*), DiGeorge Syndrome Critical Region 8 (*DGCR8*), Mitochondrial Ribosomal Protein L40 (*MRPL40*), proline dehydrogenase (*PRODH*), Solute Carrier Family 25 Member 1 (*SLC25A1*), Thioredoxin Reductase 2 (*TXNRD2*), *T10*, and Zinc Finger DHHC-Type Palmitoyltransferase 8 (*ZDHHC8*) [18]. Of these nine, the first three are believed to have an indirect effect on mitochondrial functioning, whereas the remaining six are directly involved in mitochondrial functioning. These six are maximally expressed shortly after birth, when forebrain synaptogenesis peaks [19]. Reduced gene dosage of genes involved in metabolism may lead to disrupted neuronal connectivity, synaptic signaling and neuronal metabolism [17, 18]. This, in turn, could lead to altered neurocognitive development, contributing to the various 22q11.2DS-associated cognitive and neurodevelopmental phenotypes.

Thus, studying the metabolome of the 22q11.2DS might allow for a deeper understanding of the underlying neurobiological pathways, which could also lead to better understanding of developmental disorders in general. Furthermore, studying the metabolome of individuals with 22q11.2DS may reveal how genotype and phenotype are connected in this genetic syndrome and provide insight into mechanisms underlying atypical neurodevelopment.

Here, we report and discuss results of untargeted metabolic analysis of dried blood spots derived from 22q11.2DS patients and controls, aiming to identify a metabolic “signature” for 22q11.2DS. In addition, we analyze associations between 22q11.2DS-related metabolomic patterns and two highly prevalent neurodevelopmental expressions in 22q11DS: low intellectual functioning (defined as lowest third of IQ-scores measured in sample) and autism spectrum disorder (ASD).

## Materials and methods

### Samples and procedures

This study was part of a large ongoing clinical cohort study at the University Medical Center Utrecht, the Netherlands, aiming to describe trajectories of cognitive and behavioral phenotypes in children and adolescents with 22q11.2DS [14]. All of the subjects had a molecularly confirmed 22q11.2 deletion. As part of the clinical assessments, all patients undergo routine laboratory assessment including metabolic parameters, as hyperprolinemia is highly prevalent in 22q11.2DS [20]. As part of the metabolic analyses, a dried blood spot is generated and stored as reported previously [21]. Subjects were asked to refrain from food and drinks (except water, and black tea/coffee without added sugar) 10 h before the blood test.

IQ, including parameters of full scale IQ, verbal IQ, and performance IQ, was assessed according to an age-appropriate version of the Wechsler [22]. For this study, only full scale IQ measures were used (from now on called “IQ”). ASD diagnosis was based on clinical assessment, which included the ADI-R [23] and direct clinical observation, and consistent with DSM-IV-TR diagnostic criteria [24]. Clinical assessments were conducted by a team of experienced psychiatrists and psychologists at the University Medical Center Utrecht, Department of Psychiatry.

The control group consisted of 87 individuals without 22q11.2DS from whom a dried blood spot was available. For these individuals, aged between 0 and 18 years, a routine metabolic analysis had been requested as part of a general pediatric assessment; no phenotypic or behavioral data were available for this control group. Therefore, it cannot be ensured that the control group was age- and sex-matched.

Ethical approval was obtained from the local Ethics Board (METC Utrecht, The Netherlands, 08/345) and informed consent was obtained from all participants and legal guardians prior to research procedures.

### Metabolic profiling

Sample preparation, direct infusion high-resolution mass spectrometry (DI-HRMS) and data processing was performed as previously described [25, 26]. Mass peak intensities were composed of summed intensities of isomers, as DI-HRMS cannot separate these. To compare the metabolic profiles of controls and 22q11.2DS patients, mass peak intensities were converted to *Z*-scores to normalize measurements across samples.

### Data analysis

R-software (v4.0.3) [27] was used to conduct data analysis. A flow chart for the data analysis is available in the Supplementary Materials (Supplementary Fig. 1). R code is available upon request.

Boruta as implemented in the Boruta package (v7.0.0) [28] was used to determine metabolic features informative about metabolic differences between individuals with and without 22q11.2DS. To ensure no interference of metabolites by psychotropic medication, individuals taking psychotropic medication (*n* = 12) were excluded from this step. Boruta is a wrapper around random forest (RF) analysis that selects the features that are more relevant than random probes. Tentative attributes were removed from selected variables, as these did not perform significantly better than random probes.

To visualize the extent to which 22q11.2DS patients could be distinguished from controls based on the features selected by Boruta, principal component analysis (PCA), RF analysis and logistic regression (LR) were performed. For the PCA we used the pca function from the MixOmics package (v6.14.0) [29]. RF analysis was performed using the method “rf” from the function train from the package caret (v6.0.86) [30], implemented with a 10-fold cross validation. LR was executed using the method “glm” from the train function belonging to the package caret (v6.0.86) [30]. The function roc from the package pROC was used to calculate the area under the receiver operating characteristic (AUROC) for LR (v1.17.0.1) [31].

Additionally, the Pearson correlations between the age of the patients and the first 5 principal components (PCs) of the whole metabolomics dataset available for patients were calculated to explore the potential confounding effect of the age of the patients.

The sequence of analyses described was repeated for within-patients analysis. To explore metabolic patterns associated with ASD co-occurring with 22q11.2DS, features distinguishing patients with ASD from patients without were selected. Additionally, features that significantly distinguished the third of the patients with the lowest IQ score (IQ < 62) from the third of patients with the highest IQ score (IQ > 69) were selected to explore metabolic patterns associated with IQ for 22q11.2DS. We choose to split the data into three categories instead of two, as this leads to a smaller loss of efficiency when analyzing [32].

## Results

Dried blood spots were available for 49 individuals with a confirmed 22q11.2 deletion (21 male, 42.8%). Of this group, 12 individuals used psychotropic medication (Supplementary Table 1). Subjects were aged between 11 and 27, with a mean of 16.8 (SD ± 3.3). No strong correlations (*r* < 0.4) between the age of the patients and the first 5 PCs of the whole metabolomics dataset were present (Supplementary Table 2).

The distribution of IQ scores, subdivided in full scale IQ (mean = 66.1, SD ± 10.4), verbal IQ (mean = 72.3, SD ± 12.4) and performance IQ (mean = 68.5, SD ± 11.2) was slightly skewed to the left compared to the normal distribution representative of 22q11.2DS patients as a whole [33, 34] (Supplementary Fig. 2). Out of the 49 individuals with 22q11.2DS included in this study, 22 individuals (44.8%) were diagnosed with ASD. A detailed overview of the clinical phenotype of this cohort can be found in the supplementary materials (Supplementary Table 3). A total of 1867 metabolites, and their respective isomers, were available for this analysis.

### Case–control analysis

**Fig. 1:**
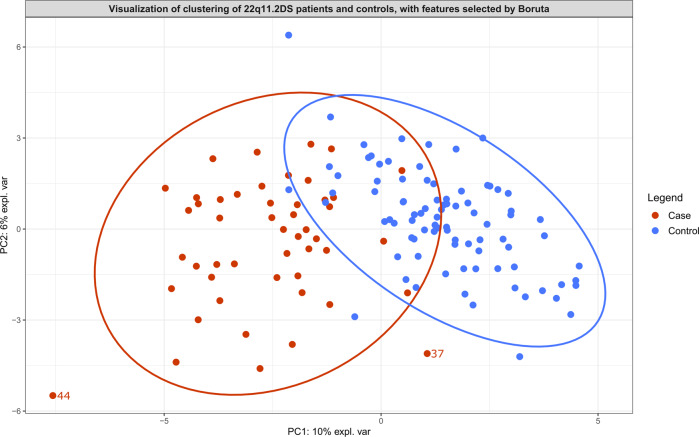
Principal component analysis score plot serving as visualization of the features selected by Boruta analysis for distinguishing 22q11.2DS patients (cases) and controls. Ellipses with a 95% confidence level serve to indicate clusters. Two patients are plotted outside of this ellipse and are labeled. More information about these patients can be found in Supplementary Table 3. No phenotypic or behavioral data were available for the control group.

Boruta identified 50 metabolites distinguishing controls and patients (Table 1; Supplementary Table 4). The PCA score plot visualizing the features selected by Boruta showed a good separation (Fig. 1). PCA contribution plots are presented in the supplementary materials (Supplementary Figs. 3–4).

**Table 1:** The metabolites that are found to be relevant, as calculated by Boruta analysis, for distinguishing 22q11.2DS patients from controls, distinguishing 22q11.2DS patients with ASD from 22q11.2DS patients without ASD and distinguishing 22q11.2DS patients with an IQ score in the lowest range (IQ < 62) from 22q11.2DS patients with an IQ score in the highest range (IQ > 69).

Metabolites distinguishing 22q11.2DS patients from controls, according to Boruta analysis.		Metabolites distinguishing 22q11.2DS patients with ASD from 22q11.2DS patients without ASD, according to Boruta analysis.	Metabolites distinguishing 22q11.2DS patients with an IQ < 62 from 22q11.2DS patients with an IQ > 69, according to Boruta analysis.
(beta-1-O-[N-(2-hydroxymethyl-3-chlorophenyl)anthraniloyl]-d-glucupyranuronic acid)	Glutamyl-Tryptophan	11′-Carboxy-alpha-tocotrienol	3-Methoxybenzenepropanoic acid
12-Ketodeoxycholic acid	Glutamyl-Tyrosine	2-Hexaprenyl-3-methyl-6-methoxy-1,4 benzoquinone	DG(14:0/14:0/0:0)
1-Methylguanosine	Glycocholic acid	4-Methylcatechol	Diethylthiophosphate
2′-Deoxyinosine triphosphate	Histamine	Bisnorbiotin	Hydroquinone
3,5-Diiodothyronine	Hydroxyphenylacetylglycine	Cer(d18:0/16:0)	Hydroxyprolyl-Isoleucine
3b,12a-Dihydroxy-5a-cholanoic acid	Hydroxyprolyl-Isoleucine	dTDP	Imidazoleacetic acid riboside
3-Hydroxyhexadecanoylcarnitine	L-Proline	Eicosapentaenoyl Ethanolamide	Se-Methylselenocysteine
3-Methoxytyrosine	LysoPE(0:0/18:2(9Z,12Z))	Estrone sulfate	Tyramine
4-Hydroxy-5-(dihydroxyphenyl)-valeric acid-O-sulfate	LysoPE(0:0/20:2(11Z,14Z))	Galactaric acid	
4-Hydroxybenzoic acid	N-(2-formyl-3-chlorophenyl)anthranilic acid	Hydroquinone	
5,10-Methenyltetrahydrofolic acid	N(6)-(Octanoyl)lysine	Leukotriene B4 dimethylamide	
5-Dodecenoic acid	N-Acetyl-L-phenylalanine	Leukotriene F4	
5-Hydroxyindoleacetic acid	PA(20:4(5Z,8Z,11Z,14Z)e/2:0)	L-Kynurenine	
5-Hydroxykynurenamine	Pantetheine		
6-Phosphonoglucono-d-lactone	Phosphoribosyl pyrophosphate		
7,8-Dihydropteroic acid	Pimelylcarnitine		
Alpha-CEHC	Propinol adenylate		
Ceramide (d18:1/12:0)	Putreanine		
cis-2-Methylaconitate	Pyronaridine		
Citric acid	Quinaprilat		
Cortolone-3-glucuronide	Retinoyl b-glucuronide		
DG(15:0/16:1(9Z)/0:0)	S-Adenosylmethionine		
d-Glucuronic acid 1-phosphate	Sphingosine		
Dihydrolipoamide	Sulfate		
Glucosylsphingosine	Thymidine 3′,5′-cyclic monophosphate		

The names of the metabolites are shown in alphabetical order. The isobars of these metabolites can be found in the supplemental materials (Supplemental Tables 4–6).

To measure the relevance of the features selected by Boruta, we performed RF analysis, resulting in an out-of-bag error (a method of estimating the prediction error for RF) of 8.4% (AUROC = 0.98, sensitivity = 0.77, specificity = 0.98), whereas logistic regression resulted in an AUROC of 0.86 (sensitivity = 0.89, specificity = 0.83).

### Within-patients analysis

**Fig. 2:**
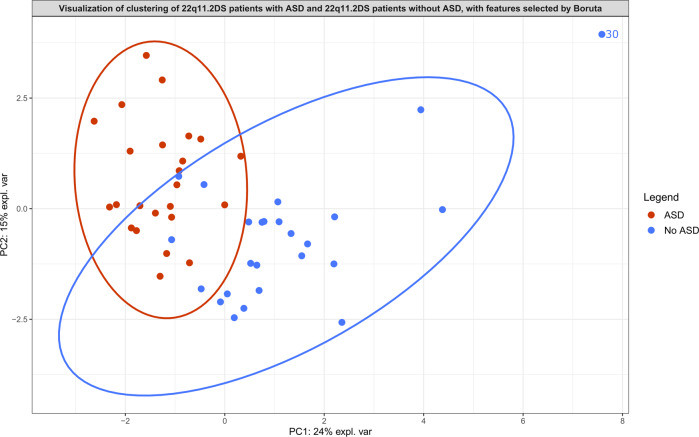
Principal component analysis score plot serving as visualization of the features selected by Boruta analysis for distinguishing 22q11.2DS patients with autism spectrum disorder (ASD) from 22q11.2DS patients without autism spectrum disorder (No ASD). Ellipses with a 95% confidence level serve to indicate clusters. One patient without ASD is plotted outside of this ellipse and is labeled. More information about this patient can be found in Supplementary Table 3.

**Fig. 3:**
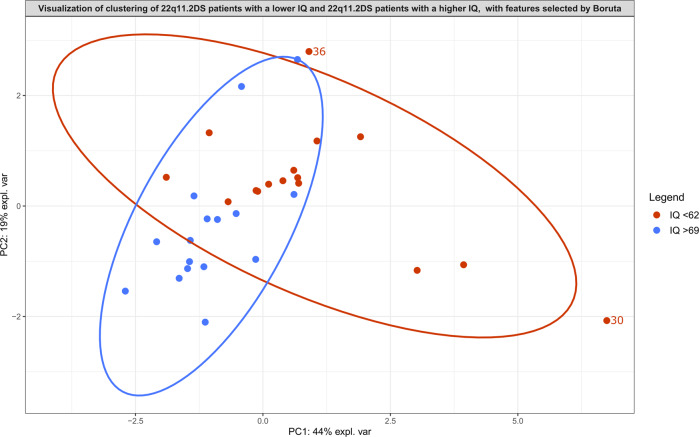
Principal component analysis score plot serving as visualization of the features selected by Boruta analysis for 22q11.2DS patients with an IQ score in the lowest range (IQ < 62) from 22q11.2DS patients with an IQ score in the highest range (IQ > 69). Ellipses with a 95% confidence level serve to indicate clusters. Two patients with an IQ score in the lowest range are plotted outside of this ellipse and are labeled. More information about these patients can be found in Supplementary Table 3.

Boruta analysis revealed 13 metabolites that significantly distinguished patients with ASD from those without ASD (Table 1 and Supplementary Table 5), and revealed 8 metabolites that significantly distinguished 22q11.2DS patients with a lower IQ (IQ < 62) from 22q11.2DS patients with a higher IQ (IQ > 69) (Table 1 and Supplementary Table 6). The PCA score plots of both within-analyses revealed relatively clear clustering (Fig. 2 and Fig. 3). PCA contribution plots can be found in the Supplementary Materials (Supplementary Figs. 5–8).

RF analysis performed to evaluate the relevance of the features selected by Boruta in distinguishing patients with ASD from those without, resulted in an out-of-bag error of 10.0% (AUROC = 0.94, sensitivity = 0.95, specificity = 0.81). The relevance of the features selected by Boruta measured with LR resulted in an AUROC of 0.73 (sensitivity = 0.67, specificity = 0.80).

RF analysis measuring the relevance of the features selected by Boruta in distinguishing patients with a lower IQ (IQ < 62) from patients with a higher IQ (IQ > 69) resulted in an out-of-bag error of 10.0% (AUROC = 0.98, sensitivity = 0.91, specificity = 0.83). LR measuring the relevance of these features selected by Boruta resulted in an AUROC of 0.75 (sensitivity = 0.67, specificity = 0.83).

## Discussion

This study performed untargeted metabolomics in dried blood spots and identified a metabolic signature for 22q11.2DS and related phenotypic expressions of ASD and IQ. The results point to potential biological mechanisms associated with 22q11.2DS and related neurodevelopmental phenotypes. This facilitates a deeper understanding of the etiology of the syndrome as well as the connection between genotype and phenotype in the context of 22q11.2DS. However, more research is required to fully interpret the broader implications of this metabolic signature.

Metabolomics is a powerful study method as metabolites and their concentrations often directly reflect biochemical activity as well as pathogenic mechanisms [35, 36]. Metabolomics allows for extensive characterization of metabolic alterations that may underlie disease etiology. Only one previous study has investigated metabolomics in a small sample of patients with 22q11.2DS (n = 11) [18]. This pilot study reported significant differences between the metabolome of children with 22q11.2DS (aged 8–15) and controls (aged 6–13). To our knowledge, our study included the largest sample size to date for any metabolomics analysis in 22q11.2DS and is the first study to report on metabolomics in adolescents and young adults with 22q11.2DS. The results reveal relevant metabolites that may guide future studies investigating pathogenic mechanisms underpinning 22q11.2DS-related phenotypes.

### Proline

Plasma proline levels are commonly increased (hyperprolinemia) in 22q11.2DS patients, occurring in 30–50% of this population [20, 37]. Therefore, it is not surprising that proline is a prominent feature of the metabolic signature. The conversion of proline to glutamate is catalyzed by the mitochondrial enzyme proline dehydrogenase [38]. This enzyme is encoded by the *PRODH* gene, which is one of the ~90 genes implicated in 22q11.2DS. Proline showed to be a significant influence in the metabolic signature distinguishing between 22q11.2DS patients and controls (Table 1 and Supplementary Fig. 3). In accordance with our findings, Napoli et al. (2015) found the metabolite proline to be significantly increased in 22q11.2DS patients compared to controls [18]. This is consistent with previous studies reporting on hyperprolinemia in patients with 22q11.2DS [20, 37]. Our secondary analyses within the 22q11.2DS patients revealed no significant differences between 22q11.2DS patients with and without ASD; or between IQ groups. This too is in line with currently available evidence demonstrating that there is no direct association between high proline levels and specific psychiatric expressions, and that additional genomic and environmental factors may be needed to produce clinical symptoms [39]. High proline levels may lower the threshold for developing neuropsychiatric disorders by indirectly affecting neuronal connectivity, synaptic signaling and neuronal metabolism [39].

### Effect of medication use

Some of the metabolites present in the metabolic signatures are associated with certain types of medication. Examples are the metabolite pyronaridine, which is used to treat malaria [40], and the metabolite quinaprilat, which is an angiotensin-converting enzyme inhibitor [41]. According to the available data, none of the participants were taking these drugs at the moment of sampling. Existing literature has not clarified this finding. It should also be noted that one of the patients took the psychotropic drug olanzapine at the moment of sampling (Supplementary Tables 1 and 3). The data of this patient, indicated by the number 30 in this study, was plotted outside of the ellipse (95% confidence level) in the PCA graphs of the observed metabolic signature of ASD and the observed metabolic signature of IQ (Figs. 2 and 3). Olanzapine has been shown to significantly alter metabolic patterns [42, 43], which may explain the observed separation of patient 30 in both Figs. 2 and 3.

### Strengths and limitations

Strengths of this study are the large sample size and thorough psychiatric phenotyping. A limitation of the study is that interpretation of results may be hampered by the fact that 22q11.2DS subjects had a fasting blood sample taken whereas for controls this was not the case. However, a 10 h fast is considered brief and insufficient to activate a full fasting response. We checked the metabolites present in the metabolic signatures for relevance to fasting pathways but none were identified. Another limitation is that the age of 22q11.2DS patients ranged from 11–27 years. However, as no correlations between age and metabolic data were identified, the influence of age is likely to be small (Supplementary Table 2).

Another limitation is that phenotypic data about the control group were unavailable﻿. Ideally, future studies would include healthy controls for whom phenotypic data are available, in order to allow for better interpretation of between-group comparisons. Furthermore, longitudinal designs are needed to investigate risk of psychiatric illness over time. The 22q11.2DS is associated with an increased risk of psychotic disorders up to 25% [9]. A longitudinal study design would enable identification of metabolic features associated with psychosis risk. Ultimately, metabolic data jointly with clinical and other data may be used for predictive modeling of disease risk and stratification.

## Conclusion

In conclusion, this study used untargeted metabolomics in dried blood spots to identify a metabolic signature for 22q11.2DS and related neurodevelopmental expressions, ASD and low intellectual functioning. By examining metabolic characteristics of 22q11.2DS we aim to detect biological mechanisms underlying these neurodevelopmental traits. Increasing our understanding of metabolic mechanisms underlying phenotypic expressions of 22q11.2DS facilitates identification of novel therapeutic targets that may ultimately lead to improved treatment strategies for patients with neurodevelopmental disorders.

## Supplementary information

Supplementary Figure 1

Supplementary Figure 2

Supplementary Figure 3

Supplementary Figure 4

Supplementary Figure 5

Supplementary Figure 6

Supplementary Figure 7

Supplementary Figure 8

Supplementary Table 1

Supplementary Table 2

Supplementary Table 3

Supplementary Table 4

Supplementary Table 5

Supplementary Table 6

## References

[1] Rauch A, Hoyer J, Guth S, Zweier C, Kraus C, Becker C (2006). Diagnostic yield of various genetic approaches in patients with unexplained developmental delay or mental retardation. Am J Med Genet A.

[2] Vorstman JA, Parr JR, Moreno-De-Luca D, Anney RJ, Nurnberger JI, Hallmayer JF (2017). Autism genetics: opportunities and challenges for clinical translation. Nat Rev Genet.

[3] McDonald-McGinn DM, Sullivan KE, Marino B, Philip N, Swillen A, Vorstman JA (2015). 22q11.2 deletion syndrome. Nat Rev Dis Prim.

[4] Blagojevic C, Heung T, Theriault M, Tomita-Mitchell A, Chakraborty P, Kernohan K (2021). Estimate of the contemporary live-birth prevalence of recurrent 22q11.2 deletions: a cross-sectional analysis from population-based newborn screening. C Open.

[5] Botto LD, May K, Fernhoff PM, Correa A, Coleman K, Rasmussen SA (2003). A population-based study of the 22q11.2 deletion: phenotype, incidence, and contribution to major birth defects in the population. Pediatrics.

[6] Kim E-H, Yum M-S, Lee B-H, Kim H-W, Lee H-J, Kim G-H, et al. Epilepsy and other neuropsychiatric manifestations in children and adolescents with 22q11.2 deletion syndrome. J Clin Neurol. 2016 10.3988/jcn.2016.12.1.85. 10.3988/jcn.2016.12.1.85 PMC4712291 26754781

[7] Guna A, Butcher NJ, Bassett AS (2015). Comparative mapping of the 22q11.2 deletion region and the potential of simple model organisms. J Neurodev Disord..

[8] Boot E, Butcher NJ, Udow S, Marras C, Mok KY, Kaneko S (2018). Typical features of Parkinson disease and diagnostic challenges with microdeletion 22q11.2. Neurology.

[9] Schneider M, Debbané M, Bassett AS, Chow EW, Fung WL, van den Bree M (2014). Psychiatric disorders from childhood to adulthood in 22q11.2 deletion syndrome: results from the international consortium on brain and behavior in 22q11.2 deletion syndrome. Am J Psychiatry.

[10] Solot CB, Knightly C, Handler SD, Gerdes M, McDonald-McGinn DM, Moss E (2000). Communication disorders in the 22Q11.2 microdeletion syndrome. J Commun Disord..

[11] Vorstman JAS, Morcus M, Duijff SN, Klaassen P, Heineman-de Boer JA, Beemer FA (2006). The 22q11.2 deletion in children: high rate of autistic disorders and early onset of psychotic symptoms. J Am Acad Child Adolesc Psychiatry.

[12] Zinkstok JR, Boot E, Bassett AS, Hiroi N, Butcher NJ, Vingerhoets C (2019). Neurobiological perspective of 22q11.2 deletion syndrome. Lancet Psychiatry.

[13] De Smedt B, Devriendt K, Fryns JP, Vogels A, Gewillig M, Swillen A (2007). Intellectual abilities in a large sample of children with Velo-Cardio-Facial Syndrome: an update. J Intellect Disabil Res.

[14] Fiksinski AM, Breetvelt EJ, Duijff SN, Bassett AS, Kahn RS, Vorstman JAS (2017). Autism Spectrum and psychosis risk in the 22q11.2 deletion syndrome. Findings from a prospective longitudinal study. Schizophr Res..

[15] Davies RW, Fiksinski AM, Breetvelt EJ, Williams NM, Hooper SR, Monfeuga T (2020). Using common genetic variation to examine phenotypic expression and risk prediction in 22q11.2 deletion syndrome. Nat Med..

[16] Fiksinski AM, Heung T, Corral M, Breetvelt EJ, Costain G, Marshall CR, et al. Within-family influences on dimensional neurobehavioral traits in a high-risk genetic model. Psychol Med. 2021; 1–9 10.1017/S0033291720005279. 10.1017/S0033291720005279 PMC9693655 33443009

[17] Meechan DW, Maynard TM, Tucker ES, LaMantia AS (2011). Three phases of DiGeorge/22q11 deletion syndrome pathogenesis during brain development: patterning, proliferation, and mitochondrial functions of 22q11 genes. Int J Dev Neurosci.

[18] Napoli E, Tassone F, Wong S, Angkustsiri K, Simon TJ, Song G (2015). Mitochondrial citrate transporter-dependent metabolic signature in the 22q11.2 deletion syndrome. J Biol Chem..

[19] Maynard TM, Meechan DW, Dudevoir ML, Gopalakrishna D, Peters AZ, Heindel CC (2008). Mitochondrial localization and function of a subset of 22q11 deletion syndrome candidate genes. Mol Cell Neurosci..

[20] Raux G, Bumsel E, Hecketsweiler B, van Amelsvoort T, Zinkstok J, Manouvrier-Hanu S (2007). Involvement of hyperprolinemia in cognitive and psychiatric features of the 22q11 deletion syndrome. Hum Mol Genet.

[21] Van Dooijeweert B, Broeks MH, Verhoeven-Duif NM, Van Beers EJ, Nieuwenhuis EE, Van Solinge WW (2020). Untargeted metabolic profiling in dried blood spots identifies disease fingerprint for pyruvate kinase deficiency. Haematologica.

[22] Wechsler D. Wechsler intelligence scale for children. San Antonio: Pearson; 2014.

[23] Lord C, Rutter M, Le Couteur A (1994). Autism Diagnostic Interview-Revised: a revised version of a diagnostic interview for caregivers of individuals with possible pervasive developmental disorders. J Autism Dev Disord.

[24] American Psychiatric Association. Washington, DC: Diagnostic and statistical manual of mental disorders. DSM-IV-TR; 2000.

[25] Haijes HA, Willemsen M, Van der Ham M, Gerrits J, Pras-Raves ML, Prinsen H (2019). Direct infusion based metabolomics identifies metabolic disease in patients’ dried blood spots and plasma. Metabolites.

[26] de Sain-van der Velden MGM, van der Ham M, Gerrits J, Prinsen H, Willemsen M, Pras-Raves ML (2017). Quantification of metabolites in dried blood spots by direct infusion high resolution mass spectrometry. Anal Chim Acta.

[27] R Core Team. R: A language and environment for statistical computing. R Foundation for Statistical Computing. https://www.r-project.org/. R Foundation for Statistical Computing; 2020.

[28] Kursa MB, Rudnicki WR (2010). Feature selection with the boruta package. J Stat Softw..

[29] Rohart F, Gautier B, Singh A, Le Cao K-A (2017). mixOmics: an R package for ‘omics feature selection and multiple data integration. PLOS Comput Biol..

[30] Kuhn M. Classification and regression training [R package caret version 6.0-88]; 2021.

[31] Robin X, Turck N, Hainard A, Tiberti N, Lisacek F, Sanchez JC (2011). pROC: an open-source package for R and S+ to analyze and compare ROC curves. BMC Bioinforma.

[32] Gelman A, Park DK. Splitting a predictor at the upper quarter or third and the lower quarter or third. Am Stat. 2012. 10.1198/tast.2009.0001.

[33] Fiksinski AM, Bearden CE, Bassett AS, Kahn RS, Zinkstok JR, Hooper SR (2021). A normative chart for cognitive development in a genetically selected population. Neuropsychopharmacology.

[34] Swillen A, McDonald‐McGinn D (2015). Developmental trajectories in 22q11.2 deletion. Am J Med Genet C Semin Med Genet..

[35] Clish CB (2015). Metabolomics: an emerging but powerful tool for precision medicine. Mol Case Stud.

[36] Gerszten RE, Wang TJ (2008). The search for new cardiovascular biomarkers. Nature.

[37] Goodman BK, Rutberg J, Lin WW, Pulver AE, Thomas GH, Geraghty MT (2000). Hyperprolinaemia in patients with deletion (22)(q11.2) syndrome. J Inherit Metab Dis.

[38] Bender HU, Almashanu S, Steel G, Hu CA, Lin WW, Willis A (2005). Functional consequences of PRODH missense mutations. Am J Hum Genet..

[39] Namavar Y, Duineveld DJ, Both G, Fiksinski AM, Vorstman J, Verhoeven-Duif NM (2021). Psychiatric phenotypes associated with hyperprolinemia: a systematic review. Am J Med Genet Part B Neuropsychiatr Genet..

[40] Croft SL, Duparc S, Arbe-Barnes SJ, Craft JC, Shin CS, Fleckenstein L (2012). Review of pyronaridine anti-malarial properties and product characteristics. Malar J.

[41] Kieback AG, Felix SB, Reffelmann T (2009). Quinaprilat: a review of its pharmacokinetics, pharmacodynamics, toxicological data and clinical application. Expert Opin drug Metab Toxicol.

[42] Newcomer J. Metabolic considerations in the use of antipsychotic medications: a review of recent evidence. J Clin Psychiatry. PMID: 17286524; 2007. 17286524

[43] Paredes RM, Quinones M, Marballi K, Gao X, Valdez C, Ahuja SS (2014). Metabolomic profiling of schizophrenia patients at risk for metabolic syndrome. Int J Neuropsychopharmacol.

