# Supplementary material for: Untargeted metabolic analysis in dried blood spots reveals metabolic signature in 22q11.2 deletion syndrome
Source: Transl Psychiatry. 2022 Mar 9;12:97. doi: 10.1038/s41398-022-01859-4 (PMC8907226; doi:10.1038/s41398-022-01859-4)
Supplement: Supplementary file 1 — Supplementary Figure 1 [file 41398_2022_1859_MOESM1_ESM.pdf]

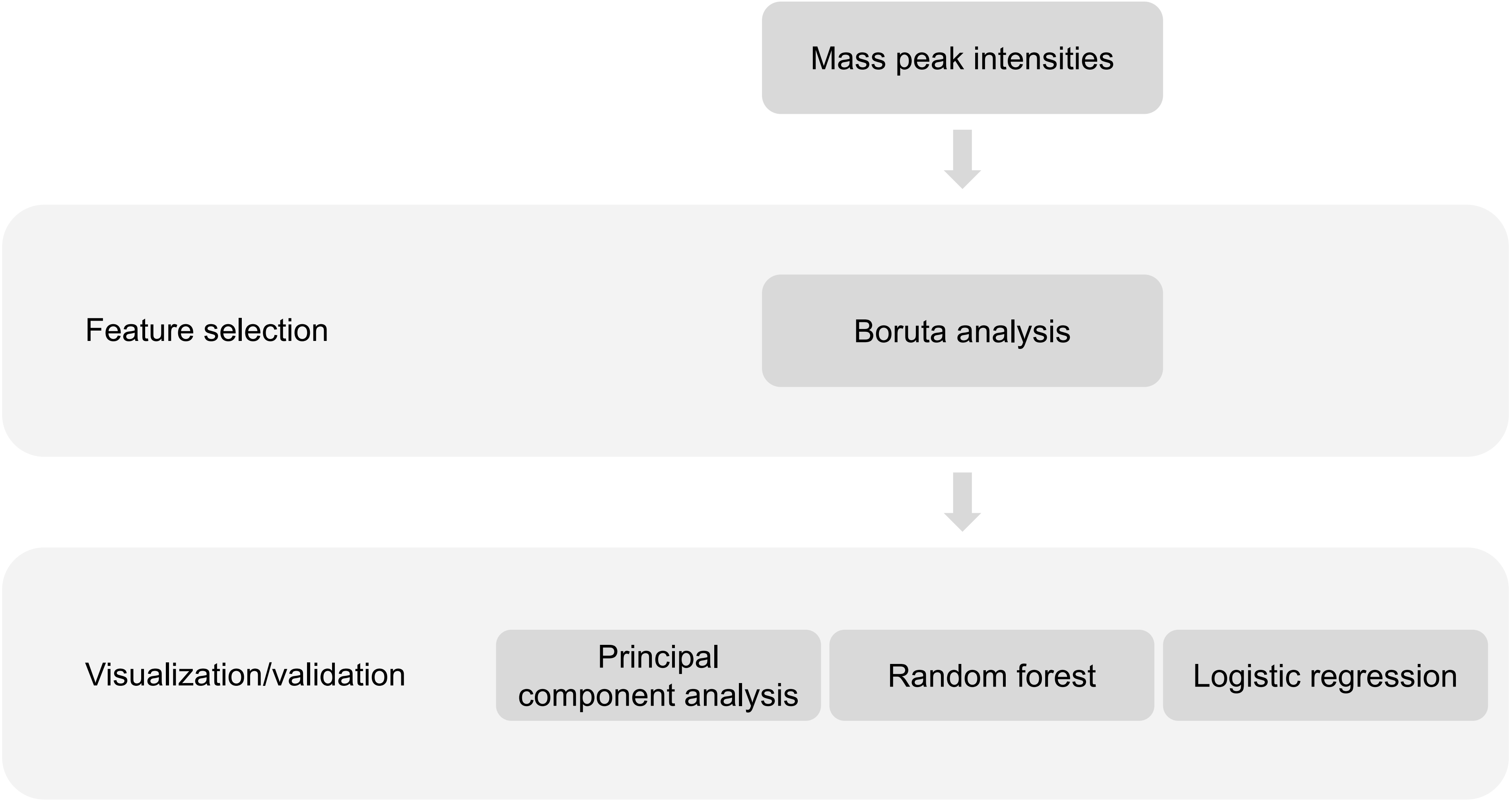
Mass peak intensities
Feature selection
Boruta analysis
Visualization/validation
Principal component analysis
Random forest
Logistic regression
