# Supplementary figures and images for: Untargeted metabolic analysis in dried blood spots reveals metabolic signature in 22q11.2 deletion syndrome
Source: Transl Psychiatry. 2022 Mar 9;12:97. doi: 10.1038/s41398-022-01859-4 (PMC8907226; doi:10.1038/s41398-022-01859-4)

**A) Full Scale IQ of 22q11.2DS patients**

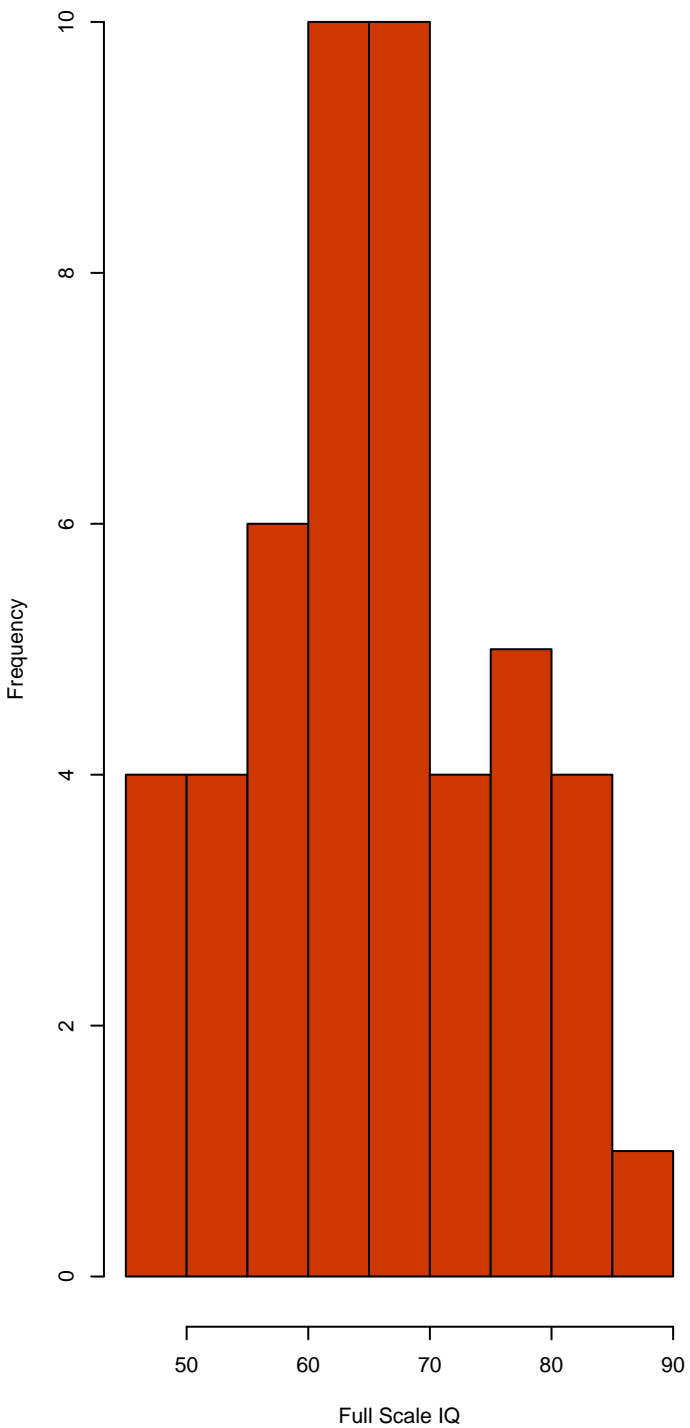

**B) Verbal IQ of 22q11.2DS patients**

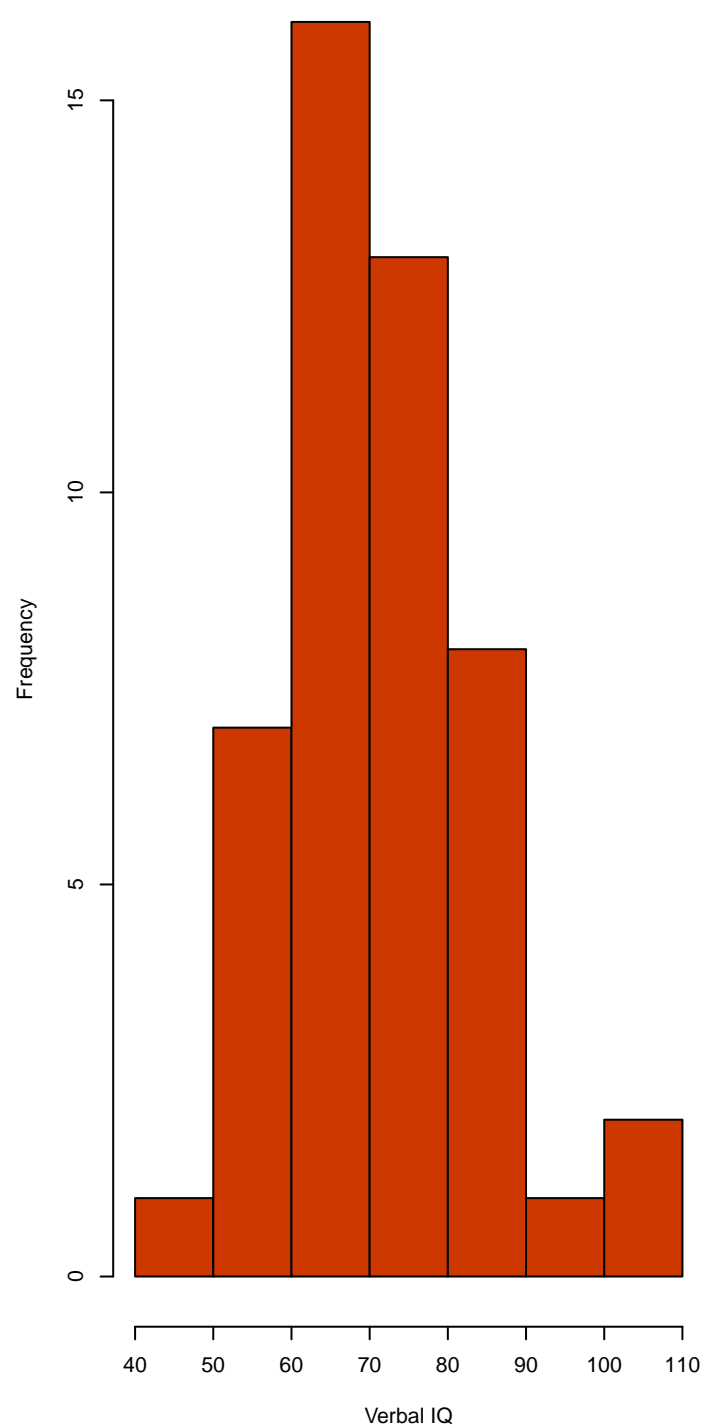

**C) Performance IQ of 22q11.2DS patients**

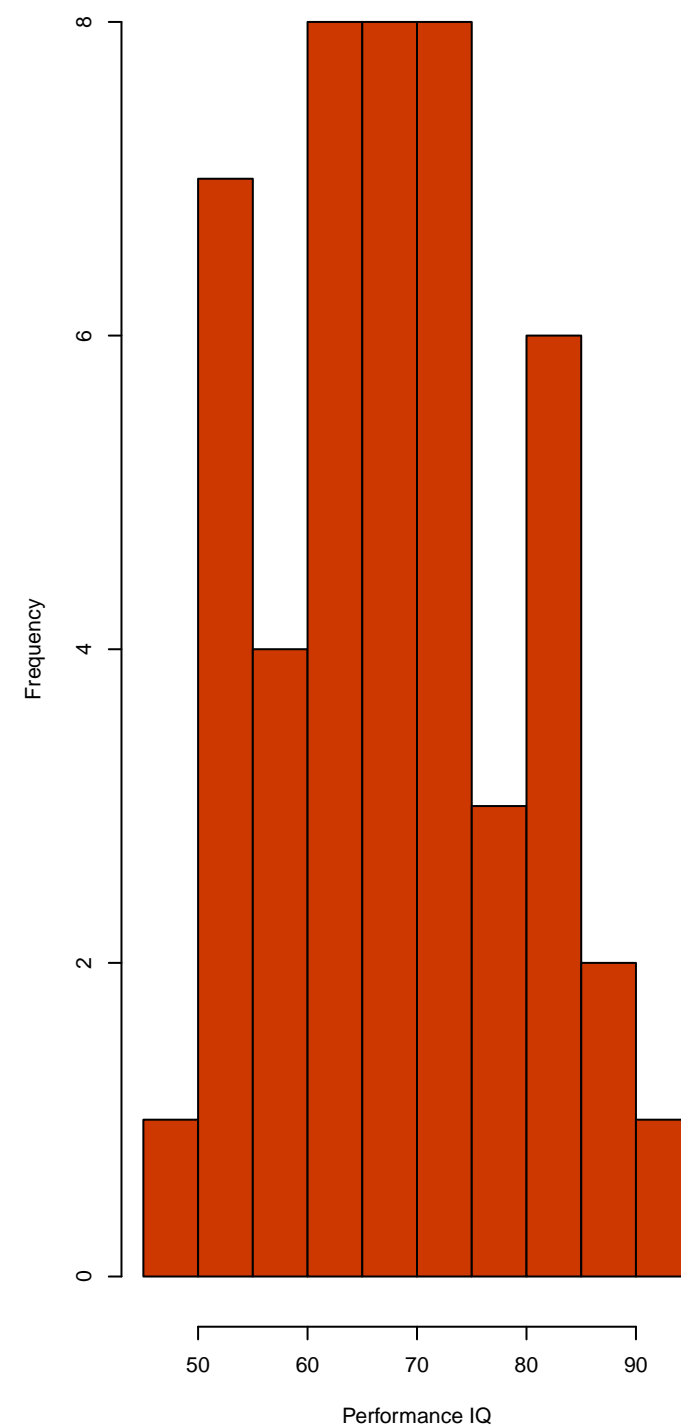

Supplement: Supplementary file 2 — Supplementary Figure 2 [file 41398_2022_1859_MOESM2_ESM.pdf]

PCA contribution plot for 22q11.2DS patients and controls; PC1

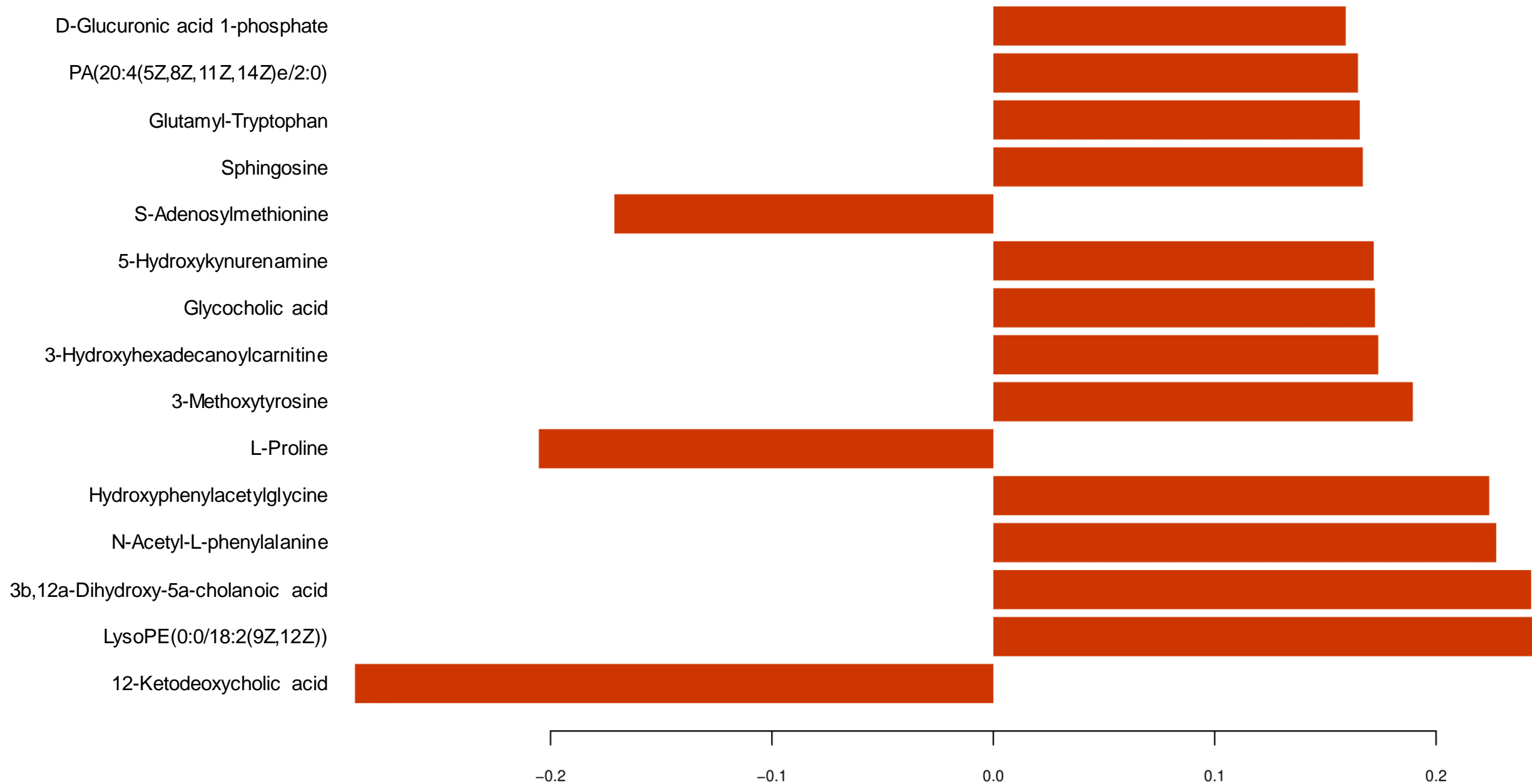

Supplement: Supplementary file 3 — Supplementary Figure 3 [file 41398_2022_1859_MOESM3_ESM.pdf]

PCA contribution plot for 22q11.2DS patients and controls; PC2

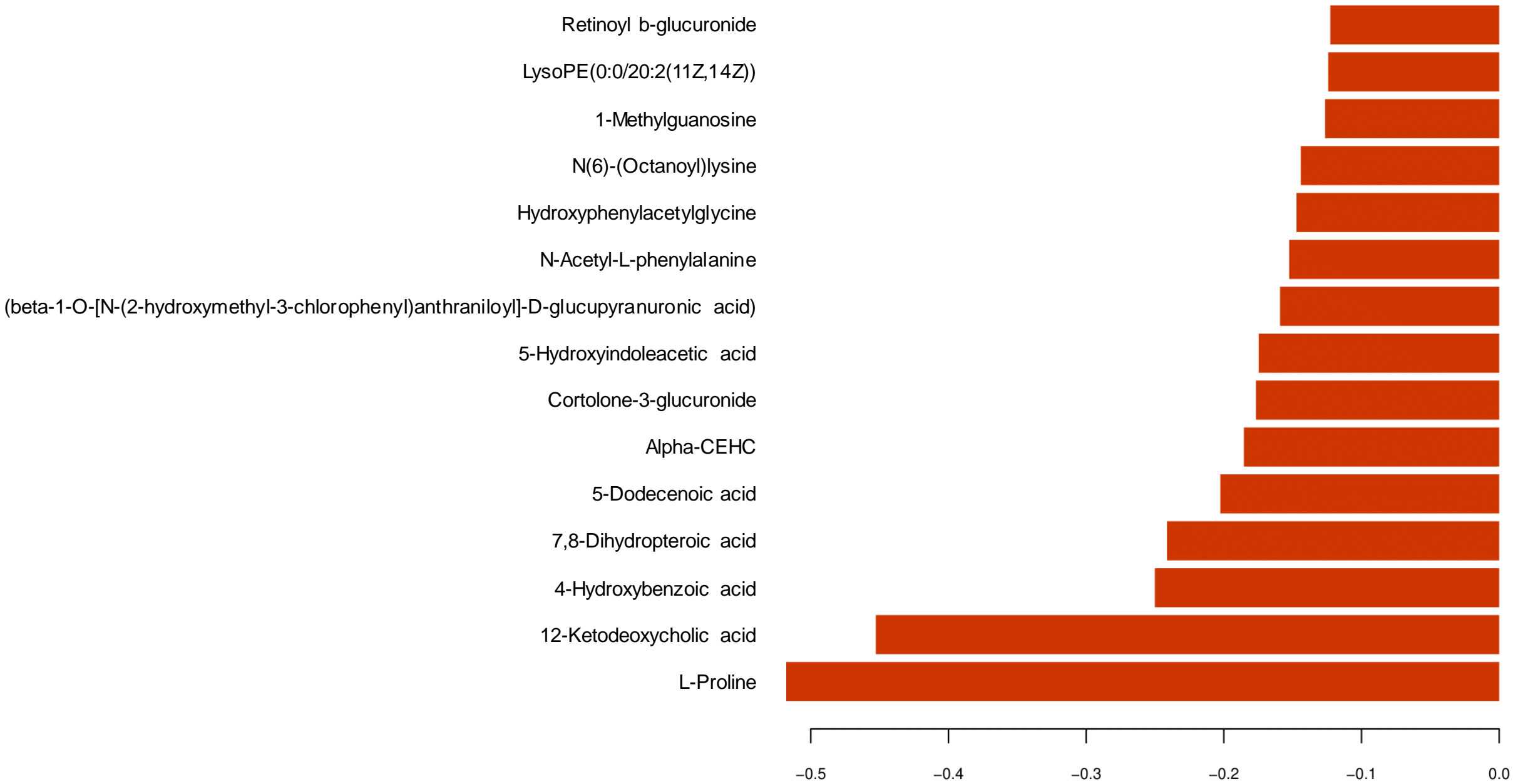

Supplement: Supplementary file 4 — Supplementary Figure 4 [file 41398_2022_1859_MOESM4_ESM.pdf]

PCA contribution plot for 22q11.2DS patients with ASD from 22q11.2DS patients without ASD; PC1

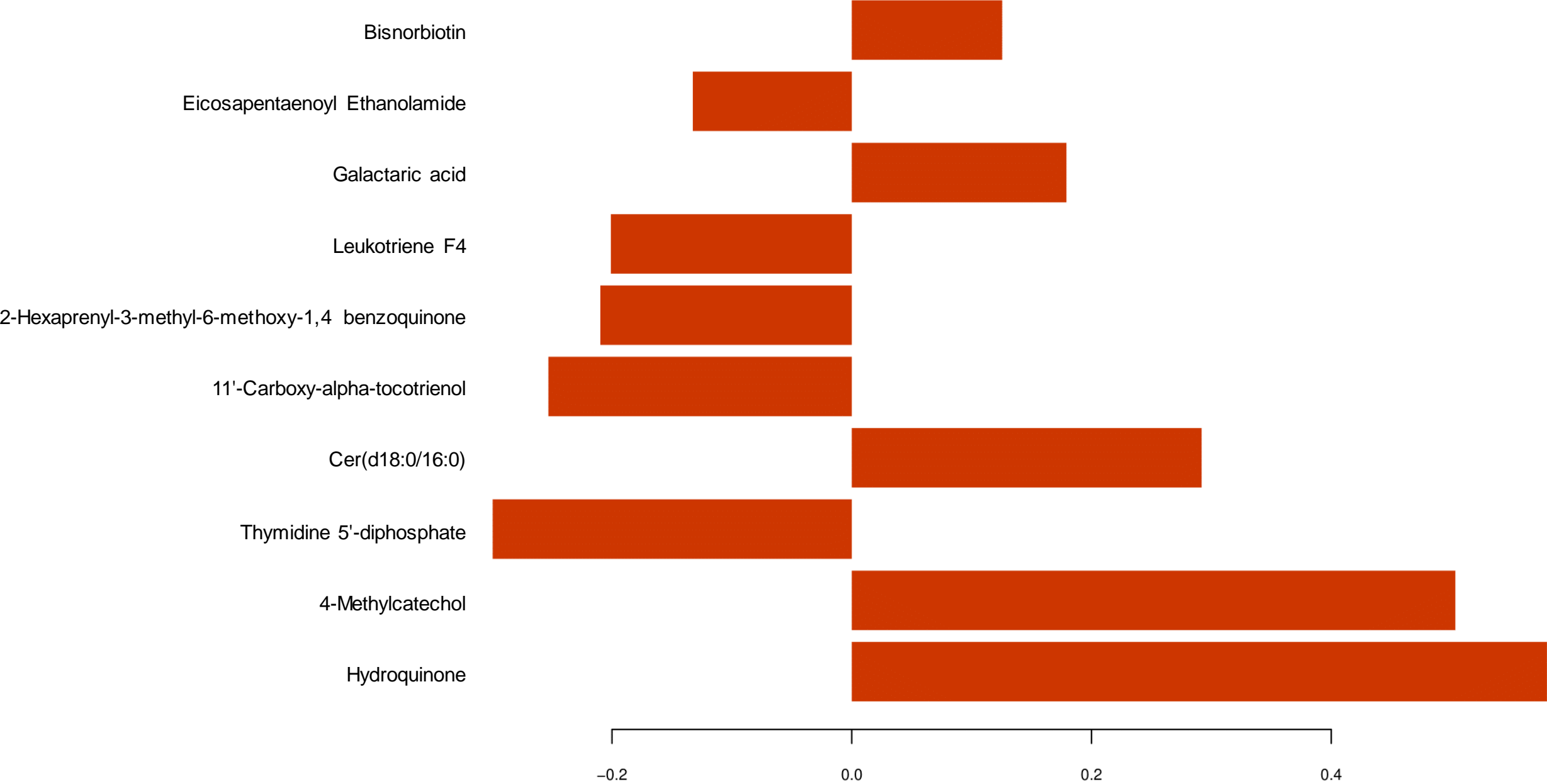

Supplement: Supplementary file 5 — Supplementary Figure 5 [file 41398_2022_1859_MOESM5_ESM.pdf]

PCA contribution plot for 22q11.2DS patients with ASD from 22q11.2DS patients without ASD; PC2

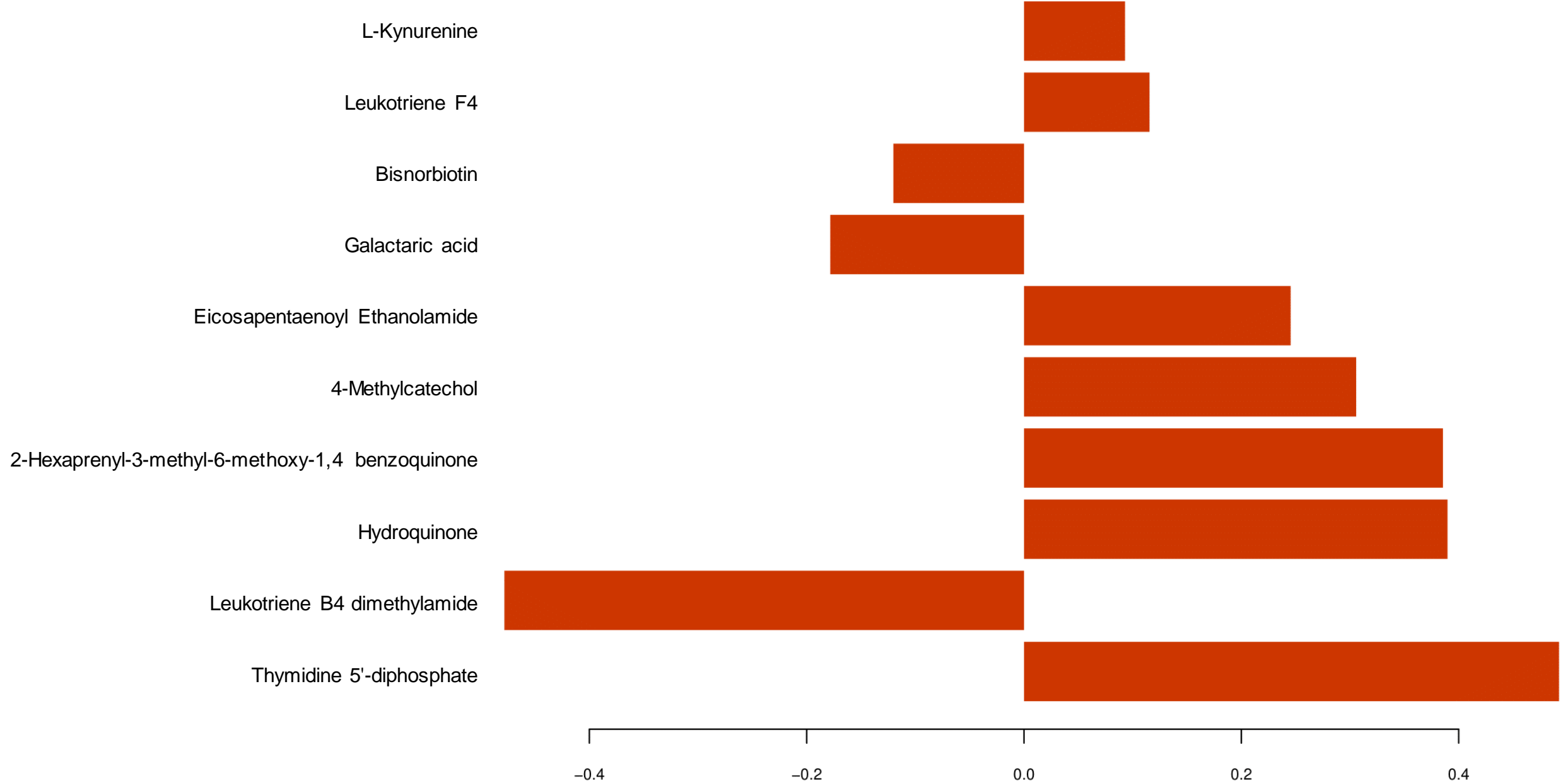

Supplement: Supplementary file 6 — Supplementary Figure 6 [file 41398_2022_1859_MOESM6_ESM.pdf]

PCA contribution plot for 22q11.2DS patients with a lower IQ from 22q11.2DS patients with a higher IQ; PC1

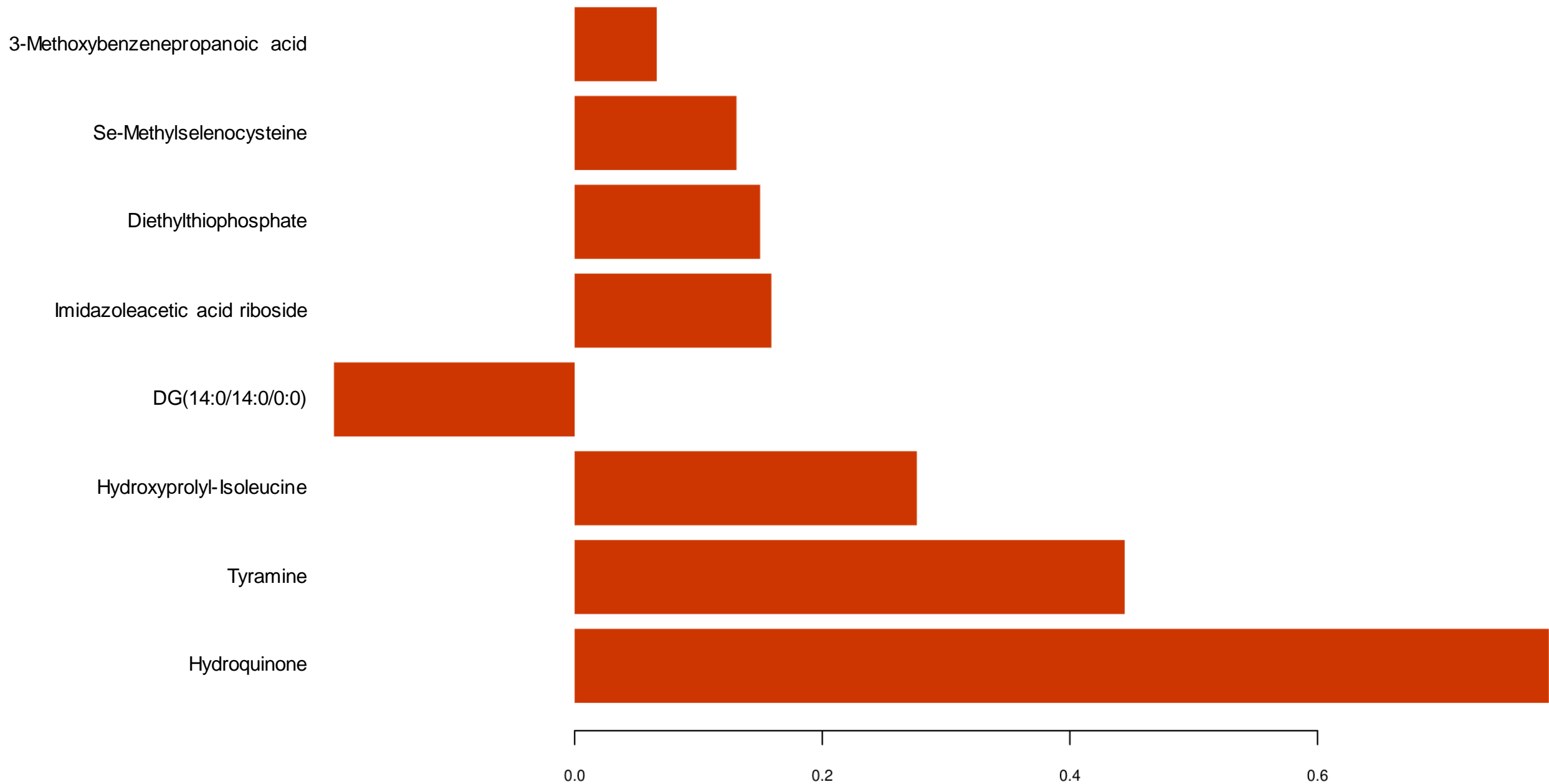

Supplement: Supplementary file 7 — Supplementary Figure 7 [file 41398_2022_1859_MOESM7_ESM.pdf]

PCA contribution plot for 22q11.2DS patients with a lower IQ from 22q11.2DS patients with a higher IQ; PC2

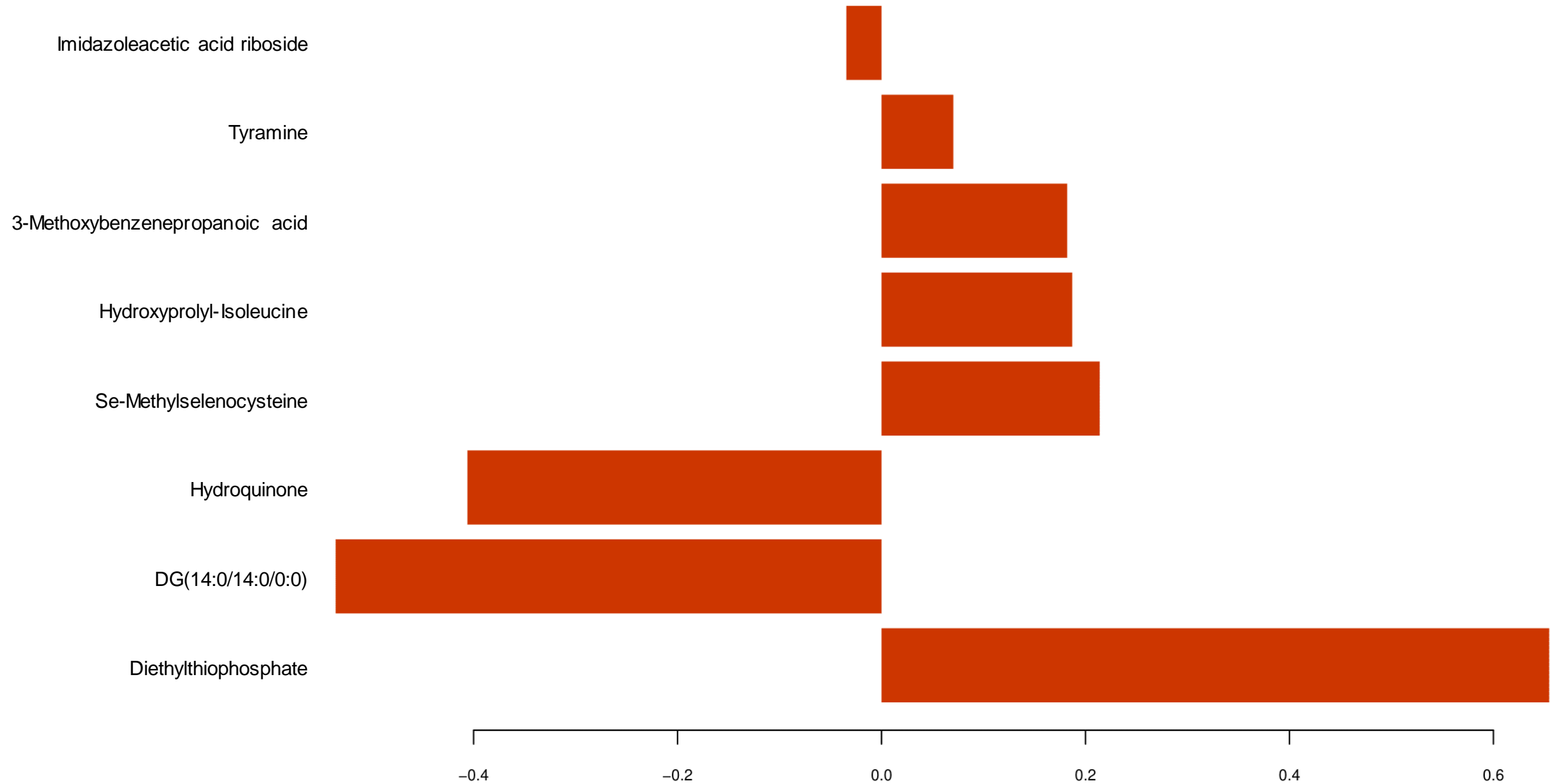

Supplement: Supplementary file 8 — Supplementary Figure 8 [file 41398_2022_1859_MOESM8_ESM.pdf]
